# Supplementary material for: A systematic approach to inserting split inteins for Boolean logic gate engineering and basal activity reduction
Source: Nat Commun. 2021 Apr 13;12:2200. doi: 10.1038/s41467-021-22404-9 (PMC8044194; doi:10.1038/s41467-021-22404-9)
Supplement: Supplementary file 2 — Reporting Summary [file 41467_2021_22404_MOESM2_ESM.pdf]

nature research

Corresponding author(s): Baojun Wang

Last updated by author(s): Mar 10, 2021

Reporting Summary

Nature Research wishes to improve the reproducibility of the work that we publish. This form provides structure for consistency and transparency in reporting. For further information on Nature Research policies, see Authors & Referees and the Editorial Policy Checklist .

Statistics

For all statistical analyses, confirm that the following items are present in the figure legend, table legend, main text, or Methods section.

n/a	Confirmed	
☐	☒	The exact sample size (*n*) for each experimental group/condition, given as a discrete number and unit of measurement
☐	☒	A statement on whether measurements were taken from distinct samples or whether the same sample was measured repeatedly
☐	☒	The statistical test(s) used AND whether they are one- or two-sided<br>*Only common tests should be described solely by name; describe more complex techniques in the Methods section.*
☒	☐	A description of all covariates tested
☐	☒	A description of any assumptions or corrections, such as tests of normality and adjustment for multiple comparisons
☐	☒	A full description of the statistical parameters including central tendency (e.g. means) or other basic estimates (e.g. regression coefficient) AND variation (e.g. standard deviation) or associated estimates of uncertainty (e.g. confidence intervals)
☐	☒	For null hypothesis testing, the test statistic (e.g. *F*, *t*, *r*) with confidence intervals, effect sizes, degrees of freedom and *P* value noted<br>*Give P values as exact values whenever suitable.*
☒	☐	For Bayesian analysis, information on the choice of priors and Markov chain Monte Carlo settings
☒	☐	For hierarchical and complex designs, identification of the appropriate level for tests and full reporting of outcomes
☒	☐	Estimates of effect sizes (e.g. Cohen's *d*, Pearson's *r*), indicating how they were calculated

Our web collection on statistics for biologists contains articles on many of the points above.

Software and code

Policy information about availability of computer code

Data collection	Image Studio v5.2; Omega Control v5.11 R4; Attune NxT Software v3.2.1, BD FACS Diva Software v6.1.3
Data analysis	Image Studio Lite v5.2; Omega MARS Data Analysis Software v3.32; PROMALS3D (http://prodata.swmed.edu/promals3d/promals3d.php); SWISS-MODEL (http://swissmodel.expasy.org/); JPred4 (http://www.compbio.dundee.ac.uk/jpred/); PyMOL v1.7.6.7; Python with packages of FlowCytometryTools v0.5.0 (http://eyurtsev.github.io/FlowCytometryTools/), FlowCal v1.3.0 (https://github.com/taborlab/FlowCal), SciPy v1.4.1 (https://www.scipy.org/), Biopython v1.76 (https://biopython.org/) and Biotite v0.20.1 (https://www.biotite-python.org/); SBOL Visual v2.2.0 (https://sbolstandard.org/docs/SBOL-Visual-2.2.pdf); BD FACS Diva Software v6.1.3; custom script deposited at tyhho/IBM (http://https://github.com/tyhho/IBM).

For manuscripts utilizing custom algorithms or software that are central to the research but not yet described in published literature, software must be made available to editors/reviewers. We strongly encourage code deposition in a community repository (e.g. GitHub). See the Nature Research guidelines for submitting code & software for further information.

Data

Policy information about availability of data

All manuscripts must include a data availability statement. This statement should provide the following information, where applicable:

- Accession codes, unique identifiers, or web links for publicly available datasets
- A list of figures that have associated raw data
- A description of any restrictions on data availability

Source data, including uncropped Western blot images and Python scripts for generating figures, are deposited to the Edinburgh DataShare [(https://doi.org/10.7488/ds/3001).]. Raw sequencing data of IBM and DIM final libraries from NGS are deposited to the Sequence Read Archive under the project accession code PRJNA678813 [https://www.ncbi.nlm.nih.gov/sra/?term=PRJNA678813]. List of constructs used in this study are detailed in Supplementary Data 1, and their sequences are available on SynBioHub76 ([https://synbiohub.org/public/Intein_assisted_Bisection_Mapping/Intein_assisted_Bisection_Mapping_collection/1).]. Representative key constructs used in this study, which allow researchers to conduct IBM of their own, are deposited at Addgene (ID 161937-161955, see

Supplementary Data 1 for details). Protein structures for analysis, including mCherry (2H5Q) [https://www.rcsb.org/structure/2H5Q], TEM-1 β-lactamase (1ZG4) [https://www.rcsb.org/structure/1ZG4], TetR (4AC0) [https://www.rcsb.org/structure/4AC0], and Ssp DnaBM86 intein (6FRH) [https://www.rcsb.org/structure/6FRH] were assessed from the Protein Data Bank[rcsb.org]77, 78.

Field-specific reporting

Please select the one below that is the best fit for your research. If you are not sure, read the appropriate sections before making your selection.

☒ Life sciences ☐ Behavioural & social sciences ☐ Ecological, evolutionary & environmental sciences

For a reference copy of the document with all sections, see nature.com/documents/nr-reporting-summary-flat.pdf

Life sciences study design

All studies must disclose on these points even when the disclosure is negative.

Sample size	By the common standard within the field, for example,in Caliando, B.J. & Voigt, C.A. Targeted DNA degradation using a CRISPR device stably carried in the host genome. Nature Communications 6, 6989 (2015), and in Nielsen, A.A. et al. Genetic circuit design automation. Science 352, aac7341 (2016), we adhered to 3 biological replicates or technical replicates done on different days per experiment, similar to what we have done previously in Wan, X., Pinto, F., Yu, L. & Wang, B. Synthetic protein-binding DNA sponge as a tool to tune gene expression and mitigate protein toxicity. Nature Communications 11, 5961 (2020). For flow cytometry we collected at least 10,000 events per sample, which was sufficient to show the entire log-normal distribution of the fluorescence of the population. This indicates that the sampling is enough. No statistical method was used to predetermine sample size.
Data exclusions	As described in Methods, candidate strains from mapping that showed strong variation between technical replicates were excluded. Later during Sanger sequencing to identify split or insertion sites, sequencing results with poor reads or those indicating mixed clones were excluded.
Replication	All experiments were performed with three replicates that were either biological or technical.
Randomization	Experiments involving biological replicates started with colony picking from an agar plate. The colonies were randomly chosen. Initial screening of naïve or sorted final libraries for insertion or bisection mapping also involved picking random colonies for characterization. Experiments involving insertion or split candidate strain characterization only sought to assess reproducibility and so had no elements of randomization. None of the experiments necessitated sample allocation into experimental groups, and this was irrelevant to our experiments because we experimented with variables one factor at a time (e.g. no induction versus induction) while keeping all other factors identical.
Blinding	We did not seek to prove or reject any particular claims or hypothesis and so blinding was irrelevant. The nature of bisection or insertion mapping does not leave room for biased revelation of split or insertion sites and therefore does not necessitate blinding.

Reporting for specific materials, systems and methods

We require information from authors about some types of materials, experimental systems and methods used in many studies. Here, indicate whether each material, system or method listed is relevant to your study. If you are not sure if a list item applies to your research, read the appropriate section before selecting a response.

Materials & experimental systems

n/a	Involved in the study	
☐	☒	Antibodies
☒	☐	Eukaryotic cell lines
☒	☐	Palaeontology
☒	☐	Animals and other organisms
☒	☐	Human research participants
☒	☐	Clinical data

Methods

n/a	Involved in the study	
☒	☐	ChIP-seq
☐	☒	Flow cytometry
☒	☐	MRI-based neuroimaging

Antibodies

Antibodies used	Primary: Anti RFP-tag, pAb, Rabbit (A00682, GenScript), THE His Tag Antibody, mAb, Mouse (A00186, GenScript), and the rabbit anti-HA (902303, Biolegend, 1:1000 diluted) antibodies; Secondary: IRDye 680RD Goat anti-Rabbit (925-68071, Li-cor) and IRDye 800CW Goat anti-Mouse (925-32210, Li-cor).
Validation	Primary antibodies were commercially available, and validation were performed by the manufacturers and documented in their certificates of analysis. In addition, in Supplementary Figure 6 and 15 there are lanes where whole-cell lysates without target proteins are loaded. The absence of bands in those lanes indicated the primary antibodies did not bind to other proteins in a non-specific manner.

For Anti RFP-tag, "Specificity" as described on manufacturer's website: "GenScript RFP-tag Antibody, pAb, Rabbit specifically reacts with fusion proteins containing RFP-tags and RFP variants, such as mCherry, tdTomato, DsRed, DsRed2, mOrange. The antibody does not react with GFP or GFP-tagged proteins."

For THE His Tag Antibody, "Specificity" as described on manufacturer's website: "THETM His Tag Antibody, mAb, Mouse recognizes C-terminal, N-terminal, and internal His tagged fusion proteins."

For anti-HA, as described in the "Application Notes" on manufacturer's website: "This antibody is effective in immunoblotting (WB) and immunoprecipitation (IP) of tagged proteins.".

For IRDye 680RD Goat anti-Rabbit, as described in "Pack Insert" document, under "Purity and Specificity": "Isolation of specific antibodies was accomplished by affinity chromatography using pooled IgG covalently linked to agarose. Based on ELISA and flow cytometry, this antibody reacts with the heavy and light chains of rabbit IgG, and with the light chains of rabbit IgM and IgA. This antibody was tested by Dot Blot and/or solid-phase adsorbed for minimal crossreactivity with human, mouse, rat, sheep, and chicken serum proteins, but may cross-react with immunoglobulins from other species. The conjugate has been specifically tested and qualified for Western blot and In-Cell Western assay applications."

For IRDye 800CW Goat anti-Mouse, as described in "Pack Insert" document, under "Purity and Specificity": "Isolation of specific antibodies was accomplished by affinity chromatography using pooled mouse IgG covalently linked to agarose. Based on ELISA and flow cytometry, this antibody reacts with the heavy and light chains of mouse IgG1, IgG2a, IgG2b, and IgG3, and with the light chains of mouse IgM and IgA. This antibody was tested by Dot Blot and/or solid-phase adsorbed for minimal cross-reactivity with human, rabbit, goat, rat, and horse serum proteins, but may cross-react with immunoglobulins from other species. The conjugate has been specifically tested and qualified for Western blot applications."

Flow Cytometry

Plots

Confirm that:

- [x] The axis labels state the marker and fluorochrome used (e.g. CD4-FITC).
- [x] The axis scales are clearly visible. Include numbers along axes only for bottom left plot of group (a 'group' is an analysis of identical markers).
- [x] All plots are contour plots with outliers or pseudocolor plots.
- [x] A numerical value for number of cells or percentage (with statistics) is provided.

Methodology

Sample preparation	Sample preparations were described in Methods: For analysis, cells were diluted in 1 × PBS with 2 mg/mL kanamycin and stored at 4°C for a minimum of one hour (5 hours post-induction) prior to acquisition, or directly acquired (24 hours post-induction). For cell sorting, cells were diluted in 1 × PBS before being passed into the cytometer.
Instrument	Analysis: Attune NxT Flow Cytometer with Attune NxT Autosampler. Cell sorting: BD BioSciences FACS Aria IIu cytometer.
Software	Attune NxT Software or raw data collection. Python with FlowCytometryTools and FlowCal for analysis. Cell sorting: BD FACS Diva Software
Cell population abundance	As described in Methods, for analysis, > 10,000 events were collected and analyzed. For cell sorting: cell sorting was typically collected on Purity mode, and usually 0.5- 1 million events were collected.
Gating strategy	Gating strategies for analysis were described in Methods. Namely, samples were gated on FCS-H and SSC-H for events between 10^3 – 10^5 arbitrary units, followed by gating on YL2-A and YL2-H between 1 to 10^6 arbitrary units. For cell sorting, cells were first gated on irregularly shaped FSC-A and SSC-A gates to exclude non-cellular materials, and then gated on boundaries defined by the previous libraries with or without induction. Gate sizes and positions were tailored to individual experiments.

- [x] Tick this box to confirm that a figure exemplifying the gating strategy is provided in the Supplementary Information.
