# Supplementary material for: A systematic approach to inserting split inteins for Boolean logic gate engineering and basal activity reduction
Source: Nat Commun. 2021 Apr 13;12:2200. doi: 10.1038/s41467-021-22404-9 (PMC8044194; doi:10.1038/s41467-021-22404-9)
Supplement: Supplementary file 3 — Description of Additional Supplementary Files [file 41467_2021_22404_MOESM3_ESM.docx]

**Description of Additional Supplementary Files**

Title: Supplementary Data 1.xlsx

Descriptions:

- **List of Constructs**
  Details of plasmid constructs used in this study, including their ID, names, descriptions, resistance markers, selection marker, reference to schematic diagram in **Supplementary Fig. 27**, and their Addgene ID (if deposited).
- **Constructs for library**Summary of constructs used in constructing the bisection and insertion libraries
- **Constructs per figure**Mappings of the samples shown in figure to the physical constructs in **List of Constructs** through the construct ID.
- **List of oligonucleotides**
  Primers used in sequencing and mapping in this study.

Title: Supplementary Data 2.xlsx

Descriptions:

- **sample_sizes**Number of data points for figures and supplementary figures where the (*n* = X) numbers cannot be conveniently reported. This is done so when data from different split/insertion candidate clones were pooled together for analyses. na: not applicable.
- **p-values**
  *p*-values in Supplementary Figure 7a, 17, 20. Owing to the large number of statistical tests performed within a single figure panel, we did not report the individual statistics and *p*-values but rather the summary statistics: n.s. not significant; **p* ≤ 0.05; ***p* ≤ 0.01; ****p* ≤ 0.001 The calculated *p*-values are provided in this table.
